# Sex Differences in Clinical Characteristics, Hospital Management Practices, and In-Hospital Outcomes in Patients Hospitalized in a Vietnamese Hospital with a First Acute Myocardial Infarction

**DOI:** 10.1371/journal.pone.0095631

**Published:** 2014-04-21

**Authors:** Hoa L. Nguyen, Duc Anh Ha, Dat Tuan Phan, Quang Ngoc Nguyen, Viet Lan Nguyen, Nguyen Hanh Nguyen, Ha Nguyen, Robert J. Goldberg

**Affiliations:** 1 Institute of Population, Health and Development, Ha Noi, Viet Nam; 2 Oxford University Clinical Research Unit, Ho Chi Minh City, Viet Nam; 3 Ministry of Health, Ha Noi, Viet Nam; 4 Viet Nam National Heart Institute, Ha Noi, Viet Nam; 5 Department of Quantitative Health Sciences, University of Massachusetts Medical School, Worcester, Massachusetts, United States of America; University of Bologna, Italy

## Abstract

**Background:**

Cardiovascular disease is one of the leading causes of morbidity and mortality in Vietnam. We conducted a pilot study of Hanoi residents hospitalized with acute myocardial infarction (AMI) at the Vietnam National Heart Institute in Hanoi. The objectives of this observational study were to examine sex differences in clinical characteristics, hospital management, in-hospital clinical complications, and mortality in patients hospitalized with an initial AMI.

**Methods:**

The study population consisted of 302 Hanoi residents hospitalized with a first AMI at the largest tertiary care medical center in Hanoi in 2010.

**Results:**

The average age of study patients was 66 years and one third were women. Women were older (70 vs. 64 years) and were more likely than men to have had hyperlipidemia previously diagnosed (10% vs. 2%). During hospitalization, women were less likely to have undergone percutaneous coronary intervention (PCI) compared with men (57% vs. 74%), and women were more likely to have developed heart failure compared with men (19% vs. 10%). Women experienced higher in-hospital case-fatality rates (CFRs) than men (13% vs. 4%) and these differences were attenuated after adjustment for age and history of hyperlipidemia (OR: 2.64; 95% CI: 1.01, 6.89), and receipt of PCI during hospitalization (OR: 2.09; 95% CI: 0.77, 5.09).

**Conclusions:**

Our pilot data suggest that among patients hospitalized with a first AMI in Hanoi, women experienced higher in-hospital CFRs than men. Full-scale surveillance of all Hanoi residents hospitalized with AMI at all Hanoi medical centers is needed to confirm these findings. More targeted and timely educational and treatment approaches for women appear warranted.

## Introduction

Previous studies examining sex differences in clinical presentation, management practices, and in the risk of developing clinically important hospital complications and dying in the setting of acute myocardial infarction (AMI) have been primarily conducted in developed countries [1]–[10]. While some studies found that women had higher in hospital case-fatality rates compared with men [1], [2], [8]–[10], other studies have not [3]–[7]. There are very few studies, however, that have been carried out in low- and middle -income countries (LMICs) [11]–[14] and more observational studies are needed to monitor and improve health outcomes in patients hospitalized with AMI [15].

Vietnam is a lower-middle income country, which has been undergoing an important epidemiological transition. The overall morbidity and mortality from Non-Communicable Diseases (NCDs) has been rising rapidly over the last two decades and the NCDs have become a major societal problem. The changing epidemiologic profile of disease in Vietnam can be attributed to changes in the size and socio-demographic characteristics of the population as well as to increases in life expectancy [16]–[19]. Cardiovascular disease (CVD) is now the leading cause of death in Vietnam, accounting for approximately one quarter of all deaths annually, and the major risk factors for CVD are either on the rise or at disturbing levels in the Vietnamese population [20].

Despite national interest in describing sex differences in disease risk, presentation, and prognosis, to the best of our knowledge no studies have examined sex differences in important clinical characteristics, hospital management, in-hospital complications, and death among adults hospitalized with AMI in Vietnam. The objectives of this study were to examine sex differences in the demographic and clinical profile, hospital treatment practices, and development of important hospital clinical complications and mortality in patients hospitalized with a first AMI during 2010 at the Vietnam National Heart Institute in Hanoi.

## Methods

### Ethics Statement

The Institutional Review Board at the Institute of Population, Health and Development approved this study. The study was conducted with a waiver of patient consent. Patient records/information was anonymized and de-identified prior to analysis.

### Study Setting

This pilot study was carried out at the Vietnam National Heart Institute (VNHI). This facility is a 250 bed- tertiary care medical center in Hanoi (2009 census = 6.5 million) which treats the majority of Hanoi residents hospitalized with acute coronary disease and other NCDs.

### Methods of Ascertainment for Newly Diagnosed Cases of AMI

Computerized printouts of patients discharged from the VNHI in 2010 with possible AMI were obtained and International Classification of Disease (ICD 10) codes for possible AMI (I20–I25) were reviewed. Cases of possible AMI were independently validated according to predefined criteria for AMI. The diagnosis of AMI was made on the basis of criteria developed by the World Health Organization which includes a suggestive clinical history, serum enzyme elevations above each hospital’s normal range, and serial electrocardiographic findings during hospitalization consistent with the presence of AMI; at least 2 of these 3 criteria needed to be present for an AMI to have occurred [21]. Residents of the city of Hanoi who satisfied these criteria were included in the present investigation. Our patient population was also classified into those with ST segment elevation AMI and non-ST segment elevation AMI (STEMI), using standard classification techniques, given current interest in this area [22]. Each of the ECG’s of patients with possible AMI was overread by a physician under guidance of a senior cardiologist.

### Data Collection

We reviewed the medical records of all patients who were admitted with discharge diagnoses suggestive of AMI and reviewed each of these patient’s charts individually. For each independently validated case of AMI and satisfying our geographic eligibility criteria, a variety of socio-demographic, clinical, and medical care related information was collected from the review of the medical record and abstracted onto a standardized case-report form by trained study physicians. Information was collected about patient’s age, sex, medical insurance coverage, duration of pre-hospital delay from the time of onset of acute coronary symptoms to hospital arrival, hospital transport by ambulance, previously diagnosed comorbidities, admission heart rate, blood pressure, serum electrolytes, serum lipid and lipoprotein levels, platelet and hemostatic factors, AMI type (ST segment elevation vs. non ST segment elevation), and location (anterior vs. inferior/posterior), receipt of effective cardiac medications prior to and during the index hospitalization (e.g., ACE inhibitors/ARBs, beta blockers) and coronary interventional procedures (e.g., PCI, CABG) during hospitalization, clinically significant in-hospital complications (e.g., heart failure, stroke, atrial fibrillation), hospital discharge status, and length of hospital stay. Atrial fibrillation (AF) included the documentation of new AF in the hospital medical record or occurrence of typical electrocardiographic changes consistent with this diagnosis. Heart failure was indicated by clinical or radiographic evidence of pulmonary edema or bilateral basilar rales with an S3 gallop. Cardiogenic shock was defined as a systolic blood pressure of less than 90 mmHg in the absence of hypovolemia and associated with cyanosis, cold extremities, changes in mental status, persistent oliguria, or congestive heart failure. We restricted our patient population to those with a first AMI based on the review of information obtained from hospital medical records.

### Data Analysis

Data were presented as percentages for categorical variables and compared between men and women using chi-square or Fisher exact tests, and median (inter quartile range- IQR) for continuous variables, and compared using Wilcoxon sum-rank tests.

Logistic regression models were used to examine sex differences in in-hospital mortality. Multivariable logistic regression models were adjusted for patient’s age, prior CVD related comorbidities, and hospital management practices that significantly differed between men and women in unadjusted analyses. All analyses were performed using STATA 11.0 (StataCorp. TX).

## Results

The study population consisted of 302 Hanoi residents hospitalized with a first AMI at the VNHI, the largest tertiary care medical center in Hanoi, in 2010. The average age of study patients was 66 years and one third were women.

### Study Population Characteristics

Women were significantly older than men (mean age: 70 years vs. 64 years) and approximately two thirds of women were 70 years or older compared with only one third of men (p<0.001) (Table 1). Two thirds of the study population were transferred from other Hanoi hospitals, and among non-transferred patients women were significantly more likely to have delayed seeking medical care after the onset of acute symptoms suggestive of AMI than men. Women were significantly less likely to have smoked (1% vs. 48%), and significantly more likely to have had hyperlipidemia previously diagnosed compared with men (10% vs. 2%). Women were also more likely than men to have had hypertension and diabetes previously diagnosed.

**Table 1:** Study Population Characteristics.

	Men (n = 201)	Women (n = 101)	p-value*
Age (mean, SD), years	64(12)	70(10)	<0.001
Age (years) (n,%)			
<60	77(38.3)	17(16.8)	<0.001
60–69	54(26.9)	21(20.8)	
70–79	47(23.4)	46(45.5)	
≥80	23(11.4)	17(16.8)	
Ethnicity (n,%)			
Kinh**	196(97.5)	100(99.0)	0.35
Minority	5(2.5)	1(1.0)	
Transferred from other hospitals (n,%)	133(66.5)	65(64.4)	0.71
Pre-hospital delay in non-transferred patients (n,%) §			
<6 hours	35(52.2)	11(30.6)	**0.035**
≥6 hours	32(47.8)	25(69.4)	
Current smoking (n,%)	97(48.3)	1(1.0)	<0.001
Cardiovascular disease related comorbidity (n,%)			
Angina pectoris	10(5.0)	2(2.0)	0.17
Atrial fibrillation	2(1.0)	2(2.0)	0.41
Diabetes	28(13.9)	23(22.8)	0.053
Heart failure	1(0.5)	0(0)	NA
Hyperlipidemia	4(2.0)	10(9.9)	**0.003**
Hypertension	112(55.7)	67(66.3)	0.08
Chest pain (n,%)	189(94.0)	93(92.0)	0.52
Acute myocardial infarction (MI) characteristics (n,%)¶			
ST segment elevation MI	140(74.9)	67(71.3)	0.52
Non-ST segment elevation MI	47(25.1)	27(28.7)	
Clinical parameters on admission (median, IQR)			
Heart rate (beats/min)	86(73–100)	87(72–100)	0.59
Systolic blood pressure (mmHg)	120(110–140)	130(110–140)	0.53
Diastolic blood pressure (mmHg)	80(70–90)	80(70–90)	0.76
Laboratory findings on admission (median, IQR)			
Cholesterol (mmol/L)	4.5(3.9–5.0)	4.7(4.2–5.5)	**0.028**
LDL (mmol/L)	2.6(2.2–3.1)	2.8(2.1–3.5)	0.37
Glucose (mmol/L)	7.1(5.6–9.2)	8.1(5.3–10.3)	0.13
eGFR (ml/min/1.73m^2^)°	71(56–88)	67(55–82)	0.19
Length of hospital stay (median, IQR), days	4(2–6)	4(2–6)	0.38

*p values from chi square or Fisher exact tests for categorical variables, and t-tests or Wilcoxon-sum rank tests for continuous variables. SD: Standard deviation; IQR: Inter quartile range.

**The Kinh people are the majority ethnic group in Vietnam, comprising 87% of the population (census 2009).

§ pre-hospital delay was defined as the duration from onset of acute symptoms suggestive of AMI to the first hospital arrival.

¶ data missing in 21 patients.

°eGFR: glomerular filtration rate was calculated based on CKD-EPI Equation.

The proportion of men and women who reported a history of heart failure, atrial fibrillation, and angina pectoris were very low in this population. Seventy three percent of study population developed a STEMI, and chest pain was reported in more than 90% of patients, equally between the sexes. On hospital admission, women tended to have higher plasma cholesterol levels compared with men. Duration of hospital stay was relatively short, and similar for both men and women (median = 4 days).

### Hospital Management Practices

During the first 24 hours after hospital admission, the utilization of aspirin and lipid lowering agents was high (>90%) as was the use of ACE inhibitors (∼70%); on the other hand, the utilization of beta-blockers was comparatively low (∼30%) (Table 2). There were no significant differences in medication utilization, both singly and in combination, between women and men, but women were less likely to have undergone PCI during the first 24 hours (50% vs. 63%). During the entire hospitalization, women were less likely to have undergone cardiac catheterization (67% vs. 82%), and PCI (57% vs. 74%) compared with men. The proportion of patients undergoing CABG surgery during hospitalization was low (1% for men and 2% for women).

**Table 2:** Sex Differences in In-Hospital Management Practices.

	Men (n = 201)	Women (n = 101)	p-value*
First 24 hours after admission (n,%)			
Aspirin	194(96.5)	94(93.1)	0.18
ACE-Is	146(72.6)	69(68.3)	0.43
Beta blockers	42(20.9)	20(19.8)	0.82
Lipid lowering agents	187(93.0)	89(89.0)	0.23
All 4 medications	38(18.9)	17(16.8)	0.66
Percutaneous coronary intervention	126(62.7)	50(50.0)	**0.035**
During hospitalization (n,%)			
Aspirin	200(99.5)	100(99.0)	0.61
ACE-Is/ARBs	173(86.1)	82(81.2)	0.27
Beta blockers	65(32.3)	26(25.7)	0.24
Lipid lowering agents	188(93.5)	90(89.1)	0.18
All 4 medications	59(29.4)	23(22.8)	0.23
Hospital cardiac procedures (n,%)			
Cardiac catheterization	165(82.1)	68(67.3)	**0.004**
Percutaneous coronary intervention	149(74.1)	58(57.4)	**0.003**
Coronary artery bypass surgery	2(1.0)	2(2.0)	0.49

*p values from chi square or Fisher exact tests.

ACE-Is: Angiotensin converting enzyme inhibitors; ARBs: Angiotensin II receptor blockers.

### In-Hospital Complications

During hospitalization, women were more likely to have developed heart failure compared with men (19% vs. 10%) (Table 3). Although there were no statistically significant differences between men and women in terms of the risk of developing other important clinical complications, women tended to develop cardiogenic shock and ventricular fibrillation/cardiac arrest to a greater extent than men, while men tended to develop recurrent angina and atrial fibrillation to a greater extent than women. The development of an acute stroke was infrequent in our study sample (Table 3).

**Table 3:** Sex Differences in In-hospital Outcomes.

	Men (n = 201)	Women (n = 101)	p-value*
Complications (n,%)			
Atrial fibrillation	4(2.0)	0(0.0)	NA
Cardiogenic shock	7(3.5)	5(5.0)	0.54
Heart failure	20(10)	19(18.9)	**0.030**
Recurrent angina	13(6.5)	2(2.0)	0.07
Stroke	0(0)	1(1.0)	NA
Ventricular fibrillation or cardiac arrest	9(4.5)	7(6.9)	0.26
In-hospital mortality (n,%)	8(4.0)	13(12.9)	**0.004**

*p-values from chi-square or Fisher exact tests.

### In-Hospital CFRs

The crude in-hospital CFR was significantly higher in women than in men (13% vs. 4%, crude OR: 3.56; 95%CI: 1.43–8.90). After adjustment for age and hyperlipidemia history, these differences remained significant (adjusted OR: 2.64; 95% CI: 1.01–6.89); after further adjustment for the receipt of PCI during hospitalization, the differences became further attenuated and no longer statistically significant (adjusted OR: 2.09; 95% CI: 0.77–5.09).

## Discussion

The results from our pilot study in Hanoi, Vietnam showed that, among patients hospitalized with a first AMI, women were older, more likely to have additional CVD comorbidities present, and were less likely to have undergone PCI during hospitalization compared with men. Women experienced higher in-hospital death rates than men, but differences in hospital death rates between the sexes narrowed after adjustment for several potentially confounding factors of prognostic importance.

During the past decade, several large studies have been conducted in LMICs examining sex differences in clinical characteristics, hospital management, and outcomes of patients hospitalized with either AMI or an acute coronary syndrome (ACS) [11]–[14]. The Chinese Registry of Acute Coronary Events (CRACE) study enrolled 1,300 patients with ACS at 12 medical teaching hospitals in China between 2001–2004 [12]. A study in Thailand included nearly 3,900 patients hospitalized with STEMI between 2001–2005 [13]. The Indian Detection and Management of Coronary Heart Disease (DEMAT) study consisted of more than 1,500 patients with suspected ACS in 10 tertiary care centers in 9 cities throughout India between 2007–2008 [14]. Recently, a study in Egypt included 1,200 patients hospitalized with AMI in 5 hospitals in Cairo and Alexandria between 2009–2010 [11].

### Study Population Characteristics

In our study, women comprised approximately one third of our study population, which is comparable to the findings reported in other studies in developed countries [7], in Thailand [13] and India [14], but higher than has been reported in China [12] and in Egypt [11]. The mean age of our study population was 66 years, which was slightly older than reported in other LIMCs including Egypt [11], China [12], Thailand [13], and India [14]. In our study, women were, on average, 6 years older than men, which is comparable with prior findings reported from China [12], Thailand [13], Egypt [11] and India [14] in which women were, on average, several years older than men.

Our results showed that women were more likely to report other CVD related comorbidities than men including hyperlipidemia, diabetes, and hypertension although some of the differences were not statistically significant likely due to our small sample size. Studies from Egypt [11], China [12], Thailand [13] and India [14] have reported similar findings. In general, the prevalence of hypertension reported was relatively similar across all studies; the prevalence of diabetes in our study was comparable to China [12], but lower than in Egypt [11] and India [14]; and the prevalence of hyperlipidemia in our study was lower than that in each of these studies. In addition, we found that women were more likely to delay to seeking medical care after the onset of symptoms suggestive of AMI compared with men. This finding is consistent with worldwide data [23], [24], [25] as well as in a recent survey among patients hospitalized with AMI in China [26]. Public educational interventions for general population are needed to encourage patients to seek medical care promptly to maximize the benefits of currently available treatments.

### In-Hospital Management

The proportion of patients who received effective cardiac medications in the present study was comparable to findings observed in China [12], Egypt [11], and India [14]; however, the use of beta-blockers was much lower, while the use of PCI in our study was higher, compared to these countries. Although the use of most proven cardiac medications was high in our study, more intensive interventions to improve the optimal use of beta-blockers should be encouraged in appropriately selected patients. On the other hand, we found that women were significantly less likely to undergo PCI both during the first 24 hours after admission and during the entire hospitalization compared with men. The lower rates of women undergoing cardiac procedures have been reported in prior studies as well [7], [13], [27]. Timely and aggressive interventions for women to narrow and close this gap remain needed to improve the short-term outcomes for women presenting with signs and symptoms of AMI in Vietnam.

### In-hospital Complications and Mortality

In our study, we found that women were more likely to have developed heart failure after hospitalization with AMI compared with men, which is in line with results from the studies in China [12] and Thailand [13]; however, the heart failure rates were higher in Thailand [13] than in our study and in China [12]. On the other hand, the study in Egypt [11] did not find sex differences in hospital complications after hospitalization with AMI.

In terms of hospital mortality, our study findings are consistent with the results of previous studies in LMICs [11], [13], [14]. The study in Thailand found that women had higher in-hospital mortality than men; however, after adjustment for patient characteristics and hospital management practices, the sex specific differences no longer remained [13]. The death rates after hospitalization for AMI were higher in Thailand (24% vs. 14%) than in our study (13% vs. 4%); the study in Thailand included patients with prior AMI and those developed STEMI only and while in our study we included the first AMI only and those with both STEMI and Non-STEMI. During a relatively similar time period, the Chinese CRACE indicated that there was no difference in in-hospital mortality between the sexes (STEMI patients: 5.6% of women vs. 7.1% men died; NSTE-ACS patients: 2.1% women vs. 1.4% men) [12]. The death rates in this study were lower than in Thailand and in our study, which may be partially explained by different settings and patient groups. The Indian DEMAT study demonstrated that there were no differences in 30-day mortality between women and men (3.0% vs. 1.8%) [14]. Recently, the study of 1,200 patients hospitalized with AMI in Egypt showed that women were more likely to have died during hospitalization in unadjusted analyses (17% vs. 9%); however, after adjustment for differences in clinical presentation and other risk factors, the higher death rates in women persisted but were no longer statistically significant [11].

In comparison with worldwide data, results from the Global Registry of Acute Coronary Events (GRACE), which included more than 26,000 patients from 14 countries between1999 and 2006, suggested that although women had slightly higher crude in-hospital mortality compared to men (4% vs. 3%), these sex differences in short-term death rates no longer persisted after adjustment for potential confounding factors [7]. The death rates reported in GRACE were lower than have been observed in most studies in LMICs [11]–[13], [27] with the exception of India [14]; this may be due to the fact that patients from the GRACE study were mainly from high income countries. On the other hand, data from the Gulf Registry of Acute Coronary Syndrome (Gulf RACE), which included more than 8,000 patients with ACS from 6 Middle East countries in 2007, indicated that women experienced a higher risk for dying during hospitalization than men (14% vs. 5%) even after adjustment for potential confounding factors [23].

There are several possible reasons for the worse in-hospital survival for women hospitalized with AMI. This may be partially explained by the fact that women are older than men when experiencing their AMI [7], [11]–[14], [23], [27], and that women are more likely than men to have additional comorbidities present, such as hyperlipidemia, diabetes and hypertension [11]–[14], [23], [27]. In addition, women were more likely to delay seeking medical care after the onset of symptoms suggestive of AMI compared with men, which might prevent them from receiving time-dependent reperfusion therapy. Furthermore, women have been shown to be less likely to be treated with effective cardiac medications [12], [13], [27] and less likely to undergo cardiac procedures compared with men [7], [13], [27]. Moreover, women were more likely than men to develop important clinical complications [12], [13]. Finally, some studies have shown that men may be more likely than women to die out-of-hospital from AMI [10], [28], which could contribute to the higher in-hospital death rates observed in women hospitalized with AMI. Future prospective studies are needed to more systematically investigate the reasons behind the poorer short-term prognosis noted in women hospitalized with AMI.

### Study Limitations

The study has several limitations. First, since the study population included only patients hospitalized at one hospital, the VNHI in Hanoi, the generalizability of our findings to patients admitted to other hospitals in Hanoi is limited and unknown. Since the sample size in the present study was small, we were underpowered to detect meaningful differences in other patient characteristics, hospital management, and hospital outcomes. Second, given the short hospital length of stay, it is important to examine whether sex differences in longer term, post-discharge mortality occurred. Third, due to the nature of the study’s retrospective design, we did not have information on several patient-associated characteristics (e.g., socioeconomic status, psychological factors, body mass index), which may have confounded some of the observed associations. We also did not have information on medication contra-indications; therefore, we likely underestimated the use of appropriate medications. Finally, because patients who died before hospitalization for AMI were not included, our findings are only generalizable to patients hospitalized with AMI.

## Conclusions

In conclusion, data from our pilot study suggest that women hospitalized for an initial AMI were older, more likely to have hyperlipidemia, and were less likely to have undergone PCI during hospitalization than men. Women were at higher risk for dying during their index hospitalization than men. Data from full-scale surveillance of all Hanoi residents hospitalized with AMI will be needed to confirm these preliminary findings. Our data also identify important opportunities for interventions designed to improve the more optimal use of effective cardiac medications in all patient groups. More aggressive and timely educational and treatment approaches during hospitalization for AMI for women are warranted to improve their short-term prognosis.
